# Comparison of voluntary and forced digital leaps in higher education – Teachers’ experiences of the added value of using digital tools in teaching and learning

**DOI:** 10.1007/s10639-022-11559-7

**Published:** 2023-01-19

**Authors:** Veera Kallunki, Nina Katajavuori, Päivi Kinnunen, Henrika Anttila, Tarja Tuononen, Anne Haarala-Muhonen, Eeva Pyörälä, Liisa Myyry

**Affiliations:** Faculty of Educational Sciences, Centre for University Teaching and Learning, University of Helsinki, Helsinki, Finland

**Keywords:** Digital tools in teaching and learning, Added value/benefits, Digital leap, Teachers’ beliefs, Higher education

## Abstract

The study examines the benefits of digital tools in teaching and learning as experienced by university teachers in two different time periods: 1) during the controlled digital leap before the Covid-19 pandemic (2017–2019) and 2) during the emergency imposition of remote teaching in response to the lockdown aimed at containing the Covid-19 pandemic (2020). Teachers in different academic fields at a large multidisciplinary Finnish university (N1 = 303 and N2 = 265) responded to two open-ended questions as part of a broader questionnaire. The research identified four kinds of benefits related to digital teaching: (1) practical and administrative and (2) independence of time and place, implying practical and technical benefits; as well as (3) enhancing learning and (4) developing teaching, which are primarily pedagogical benefits, related to teaching and learning. Voluntary and forced digital leaps provided different kinds of consequences in teaching and learning. Digital tools generally provided practical and technological benefits for teaching and learning during the controlled digital leap, but they also had positive pedagogical effects. The forced digital leap, in turn, demonstrated the value of time-and-place-independent online teaching and learning. It also exposed differences among academic fields in how teachers experienced the benefits of using digital tools. Support of and training for university teachers should take into account the different needs of faculties and disciplines, and promote dialogue between pedagogical and technological interests.

## Introduction

The use of various digital tools in higher education has gradually become established during the first two decades of the twenty-first century, but it has been greatly accelerated by the ongoing Covid-19 pandemic (see e.g., García-Morales et al., 2021; Son et al., 2021; Myyry et al., 2022). Even before the pandemic, however, several teaching-development projects at universities in Finland emphasised the need to expand and support digitalisation and the use of information and communication technologies (e.g., Jääskelä et al., 2014; Kauppinen & Malmi, 2017; Malmi et al., 2018). Many of these university-wide projects were based on university strategies and received support and funding from university. Universities worldwide have been integrating teaching platforms as well as applications that support active learning since the beginning of the twenty-first century (see e.g., O’ Shea et al., 2015; Roddy et al., 2017; Serrano et al., 2019).

Initially, the digitalisation of teaching and learning in higher education meant replicating or supplementing face-to-face teaching in an online environment (Baran et al., 2011; Kirkwood & Price, 2014). Online teaching and learning environments were thought of as technological support for on-site teaching and as a repository of learning materials.

Gradually, new pedagogical challenges and opportunities for online teaching have emerged, together with different pedagogical views in an increasingly digital environment. These views include: 1) student engagement and interaction in online courses (Kilgour et al., 2019; Northcote et al., 2015); 2) different ways of learning via online technologies in different disciplines (Baran et al., 2011); 3) the teacher’s changing role in online contexts (Adnan, 2018; Baran et al., 2011); 4) the teacher’s developing digital competence (Amhag et al., 2019; Myyry et al., 2022); and 5) various new teaching approaches (e.g. blended learning/teaching and the online flipped classroom approach (Garrison & Kanuka, 2004; Hew et al., 2020; Rasheed et al., 2020). Thus, research has revealed how the use of digital tools challenges standard teaching practices and puts pressure on teachers to develop new, meaningful digital pedagogy in online learning environments. Thus far, little is known about teachers’ perceptions of the potential added value of digital tools in their teaching, or of the pedagogical rationale behind their use.

## The benefits of using digital tools in higher education

Digital tools are used in various ways for many pedagogical purposes, such as supporting collaborative learning and knowledge building (Deng & Tavares, 2013; Häkkinen & Hämäläinen, 2012), facilitating student understanding by means of visualisation, for example (e.g., Sorva et al., 2013), giving students feedback and monitoring their learning progress (Jääskelä et al., 2017) and implementing online exams and learning assessment (Marcelo & Yot-Domínguez, 2019; Myyry & Joutsenvirta, 2015). In general, it is believed that educational technology enhance**s** the design of student-centred learning environments (Hannafin & Land, 1997; Ottenbreit-Leftwich et al., 2010; Reigeluth, 2014). However, as recent studies have shown, teachers tend to use digital tools for more practical course-related purposes such as administration, handling teaching material and communicating (Amhag et al., 2019; Brady & O’Reilly, 2020; Bond et al., 2018). Moreover, digital tools are frequently used to support a teacher-focused approach whereby the student is considered a receiver of information, and student-centred learning is not promoted (Bond et al., 2018; Marcelo & Yot-Domínguez, 2019). Digital tools are used less frequently to enhance student engagement or to monitor performance, for example (Brady & O’Reilly, 2020). Brady and O’Reilly (2020) identified three main reasons for using digital learning management systems (LMS) in teaching: 1) to store materials, 2) to manage assessments and 3) for communication, of which the first was the most common. On the other hand, previous studies have reported inter-disciplinary differences in how teachers use digital tools: for assimilative and assessment activities in the social sciences, to enhance experiential learning activities in engineering and architecture, and to promote communicative learning in health sciences **(**for example Liu et al., 2020; Marcelo & Yot-Domínguez, 2019).

**Fig. 1:**
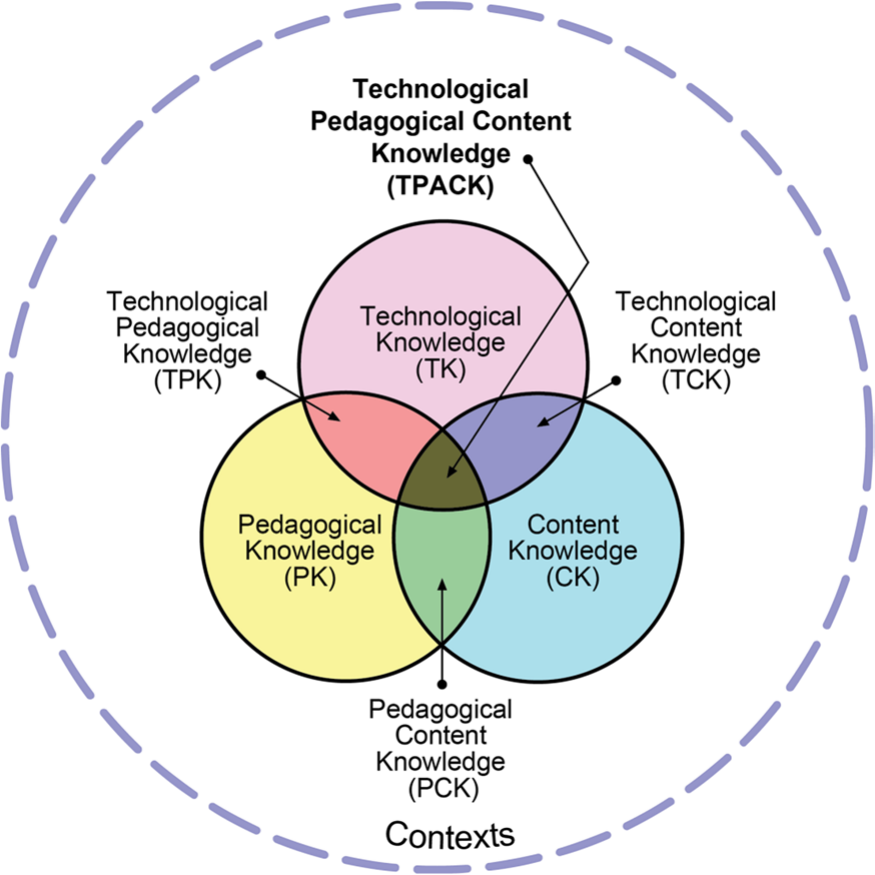
The TPACK framework: reproduced by permission of the publisher, © 2012 by tpack.org

However, less is known about why teachers choose to use digital tools in their teaching, and what the perceived benefits are. Their increasing role in teaching and learning opens up excellent opportunities for research into how teachers in higher education use them in practice (see e.g., Jääskelä et al., 2017). Teachers’ pedagogical thinking directs the choices that teachers make in online contexts, e.g**.** how they justify the use of digital tools to enhance teaching and learning. The Technological Pedagogical Content Knowledge (TPACK) framework (Fig. 1) developed by Mishra and Koehler (2006) facilitates both analysis and understanding of the pedagogical thinking of teachers in online contexts. The framework combines various aspects of teaching, such as the given content and the pedagogical choices the teacher makes with the educational technology to be used in the classroom to support teaching and learning. These three aspects of teaching are represented by three types of teacher knowledge in the TPACK framework (Fig. 1). First, content knowledge (CK) refers to domain-specific knowledge of focal facts and concepts, for example, and an understanding of how the subject knowledge is structured. Second, pedagogical knowledge (PK) includes generic knowledge of how to teach or to create a learning environment that scaffolds student learning. Third, technological knowledge (TK) refers to the competences related to different technologies and how to operate with them. It is at the four intersections of these knowledge types that teachers combine their different knowledge and skills. First, pedagogical content knowledge (PCK) refers to the teaching of content/subject matter to groups of students and deciding how best to scaffold student learning; it integrates formal knowledge and the teacher’s experimental knowledge. Second, technological content knowledge (TCK) lies at the intersection of content knowledge and technological knowledge, and focuses on issues such as the teacher’s understanding of what kinds of representations of the content technology affords. Third, technological pedagogical knowledge (TPK) comprises the teacher’s understanding of the capabilities of various technologies that might be used in educational settings. Finally, technological pedagogical content knowledge (TPACK) combines the teacher’s content, pedagogical and technological knowledge. According to Mishra and Koehler (2006, 1017), TPACK describes how “thoughtful pedagogical uses of technology require the development of a complex, situated form of knowledge.” The knowledge types, and especially their intersections, represent the different skills required for the effective use of digital tools in teaching. Figure 1 below depicts the knowledge types and their intersections.

## The benefits of using digital tools in teaching and learning—teachers’ views

To understand what motivates teachers to use digital tools, it is necessary to know what they perceive to be the benefits (over the costs) of exploiting educational technology. According to Compeau and Higgins (1995), the main driving force in changing the behaviour of individuals is the belief that the change will result in valued outcomes. For instance, as Amhag et al., (2019) state, if teachers are to be motivated to utilise technology in their classroom practice, they have to recognise its added, or surplus, value for their teaching, and to believe it will have some positive effects.

Perceived usefulness and ease of use are variables used in many studies on the acceptance of information technology (Davis, 1989). The former refers to the degree to which technology use might enhance job performance, and the latter to the degree to which its use might be free of effort. Outcome expectations, namely the extent to which certain behaviour is expected to lead to valued outcomes, are strong antecedents of technology use. According to Bandura’s (1986, 1997) social cognitive theory, outcome expectations comprise three components: performance outcomes, self-evaluative outcomes and social outcomes. In the context of educational technology, the first of these refers to beliefs that using technology enhances teaching effectiveness. Self-evaluative outcome expectations, in turn, refer to achieving personal satisfaction from using technology in teaching, and social outcome expectations to the belief that one’s peers would look favourably upon its use. Niederhauser and Perkmen (2010) studied technology-outcome expectations among pre-service teachers and confirmed the three factors described in Bandura’s (1986, 1997) theory. However, Amhag et al., (2019) claim that the first step is for teachers themselves to identify the surplus pedagogical value of educational technology for teaching and learning. Alternatively, one could analyse the perceived added value via the TPACK framework (Fig. 1) in terms of identifying to which components the expected outcomes are related. Such an analysis could, for example, reveal a more pedagogical or technological/practical orientation to using digital tools that positions the teacher’s experienced added value in different segments of the framework.

In general, experienced added value is closely related to the teacher’s pedagogical beliefs. Teacher beliefs could be described as educational opinions or tacit assumptions about teaching and learning that affect the organisation of teaching in terms of choosing the teaching methods or pedagogical tools that facilitate the achievement of learning goals (Handal et al., 2013; Jääskelä et al., 2017). In the case of online teaching, or using digital tools in on-site teaching, the teacher’s pedagogical beliefs concern the value of digital tools in student learning (Polly et al., 2010). Kim et al. (2013) highlight the need to understand teachers’ beliefs about technology integration, as well as their perceptions concerning the value of using digital tools in teaching and learning. In addition, as Nelson et al. (2019) point out, the experienced value of digital tools relates to technology training, such that teachers are made aware of the potential benefits of using them. However, the mere use of digital tools in teaching and learning does not guarantee improved educational practices, and may also have unanticipated consequences. As Liu et al. (2020) point out, therefore, future research should recognise the multidimensional aspects of using digital tools in teaching. Some teachers, for example, may think that their use has no value in, or is even detrimental to teaching and learning, and this is reflected in their negative attitude towards them (Ertmer et al., 2012; Liu et al., 2020; Mama & Hennessy, 2013).

Mei et al. (2019) studied higher education teachers’ utilisation of digital learning tools in classroom and their grounds of utilisation such technology. The sample was five participants from two different campuses. The participants were selected by a purposive sampling in order to focus on more than average experienced users of digital learning tools. In Jääskelä et al.’s (2017) research, 18 university teachers from various disciplines who had voluntarily joined a network program facilitating and supporting the use of ICT for teaching and learning was studied. The study (2017, 200) focused on “university teachers’ pedagogical beliefs and value beliefs regarding ICT use in higher education”, and “the relationship between teachers’ pedagogical aims, the practices they value as important in promoting learning, and their beliefs regarding ICT use for that purpose”. However, as both articles state, there is a need for further research on the use of digital tools among teachers in higher education (Jääskelä et al., 2017; Mei et al., 2019). More specifically, earlier studies have called for wider surveys with more respondents to get a more comprehensive picture, a focus on the added value of such tools and taking also students’ perception into account (Jääskelä et al., 2017; Mei et al., 2019; see also Amhag et al., 2019). It would be interesting to know, for example, what added value or benefit teachers in higher education think they would derive from using digital tools in their teaching, and how it could affect student learning (Amhag et al., 2019; Jääskelä et al., 2017; Mei et al., 2019; Rytkönen, 2014). Such arguments could shed light on how teachers in different academic fields of higher education think pedagogically when planning and implementing their teaching, as well as on the extent to which teachers emphasize their own benefits compared with their students’ benefits. Given the rapid worldwide digital leap in higher-education teaching and learning during the Covid-19 pandemic, as well as the recognised pedagogical pressure to change or adjust teaching when it is digitalised, this question is highly topical.

## The aim of the study

The aim of this study is to construct a picture of the benefits of digital teaching and learning tools as experienced by university teachers at a big multidisciplinary university (the University) over two different time periods. A further interesting question to be explored is whether teachers' experiences differ if the change is attributable to institutional encouragement to digitalise teaching as opposed to the enforced use of digital tools as happened during the Covid-19 pandemic. As experts have argued, the forced transition to digital environments during the pandemic differed dramatically from “online learning”, which is why a new term, namely emergency remote teaching, came into use (Bond et al., 2021; Hodges et al., 2020; Rapanta et al., 2020; Trust & Whalen, 2020). Thus, the aim is on one hand to find answers to questions concerning changes/differences in benefits before and during the Covid-19 pandemic. On the other hand, we are interested in differences among teachers depending on the discipline, and differences in teachers’ perceptions of benefits for their students and for themselves over the research period. The two datasets, therefore, offer an excellent opportunity to compare the experienced benefits of using digital tools in teaching and learning in these two very different situations. Our hypothesis is that teachers’ recent experiences of remote teaching also differ from their earlier experiences of online learning.

The research questions are as follows:

*How do university teachers perceive the benefits of using digital tools in teaching and learning at a multidisciplinary university?*

- *How do university teachers perceive the benefits of using digital tools in teaching and learning at a multidisciplinary university before and during the Covid-19 pandemic? (SQ1)*
- *What disciplinary differences are there in the teachers’ perceptions?* (SQ2)
- *What differences are there in the teachers’ perceptions of benefits for their students and for themselves? (SQ3)*

**Fig. 2:**
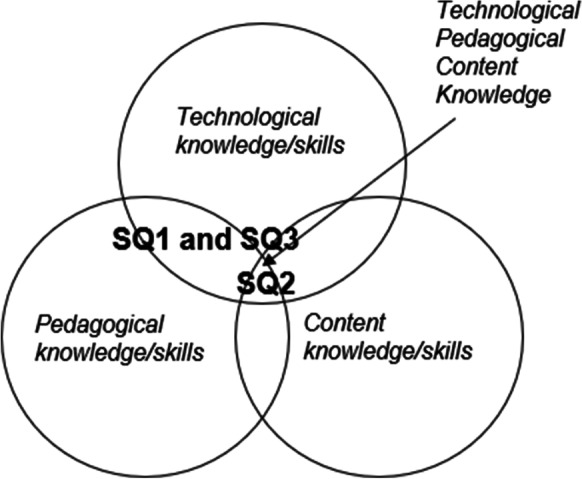
The positioning of the sub-questions (SQ1, SQ2 and SQ3) in the TPACK framework

Within the TPACK framework, our study is positioned at the intersections of the three knowledge/skills aspects of teaching (see Fig. 2). Sub-questions 1 and 3 (SQ1 and SQ3) are targeted mainly at the intersection of pedagogical knowledge and technological knowledge, so-called technological pedagogical knowledge (TPK). Content knowledge is also included in sub-question 2 (SQ2), which meant a shift in focus to the centre of the framework, namely technological pedagogical content knowledge (TPACK), in which all three aspects of teaching overlap.

## Material and methods

### Context

This study was carried out at a large multidisciplinary, research-intensive university in Finland, with approximately 4,000 teachers and researchers and 35,000 students in eleven faculties. The University invested in digital learning and teaching in 2017–2020, making the so-called digital leap by including digital learning in its strategic goals. The aim was to promote a versatile and pedagogically meaningful use of online learning by replacing traditional methods with open and digital learning environments to support students in their role as active agents. As part of this process, the university has also offered teachers support and education for digitalisation, courses in university pedagogy as well as personal help in digitising their teaching and assessment practices. (Kuuppelomäki et al., 2021) In the spring 2020 the controlled digital leap changed into a forced digital leap because of the Covid-19 pandemic. This study includes data from both time periods. This temporal placing of the two rounds of data collection, the first during the controlled digital leap at the University just before the Covid-19 pandemic, and the second during the emergency period of remote teaching during lockdown, offers a unique opportunity to examine the added value/benefits of using digital tools in teaching and learning experienced by teachers in higher education.

### Data and procedure

This research is based on two cross-sectional data sets: Data 1 was collected in 2017–2019, when digitalisation was included in the university’s strategic plan, and Data 2 in spring 2020, at the beginning the COVID-19 pandemic. In both cases potential participants were sent a link to an online questionnaire via email, and one follow-up reminder. Participation was voluntary and no compensation was provided. The Data 1 set was collected among five faculties in four phases during the period of strategic change: science (autumn 2017), arts and theology (spring 2018), education (autumn 2018) and medicine (spring 2019) (Table 1). Despite the temporal variation, it is reasonable to assume that the situation was similar in all faculties given the ongoing strategic emphasis on digitalisation and digital learning. A total of 303 teachers in higher education filled in the online questionnaire in autumn 2017/spring 2019. According to university statistics on teaching and research staff, the invitation to participate was sent to approximately 1,660 staff members, and the approximate response rate was 18 per cent.

**Table 1:** Data 1: descriptive information on the demographic variables according to the academic field

Data 1		Humanities	Educational Sciences	Medicine	Science	Theology	Total
		n (%)	n (%)	n (%)	n (%)	n (%)	n (%)
Gender	Female	50 (59%)	38 (69%)	34 (63%)	28 (35%)	14 (50%)	164 (54%)
	Male	29 (34%)	15 (27%)	20 (37%)	50 (62%)	14 (50%)	128 (42%)
	Other/missing	6 (7%)	2 (4%)	0 (0%)	3 (4%)	0 (0%)	11 (4%)
	Total	85	55	54	81	28	303
Age	20–30 years	5 (6%)	1 (2%)	2 (4%)	7 (9%)	2 (7%)	17 (6%)
	31–40 years	29 (34%)	11 (20%)	10 (19%)	21 (26%)	7 (25%)	78 (26%)
	41–50 years	22 (26%)	23 (42%)	17 (32%)	25 (31%)	10 (36%)	97 (32%)
	51–60 years	23 (27%)	16 (29%)	14 (26%)	18 (22%)	9 (32%)	80 (26%)
	over 61 years	5 (6%)	4 (7%)	11 (20%)	10 (12%)	0 (0%)	30 (10%)
Pedagogical training	None	18 (21%)	1 (2%)	4 (7%)	29 (36%)	1 (4%)	53 (18%)
	Basic studies (1–25 credits)	41 (48%)	5 (9%)	37 (69%)	36 (44%)	15 (54%)	134 (44%)
	Intermediate-level studies (25–59 credits)	4 (5%)	3 (6%)	3 (6%)	6 (7%)	5 (18%)	21 (7%)
	60-credit study module	4 (5%)	7 (13%)	3 (6%)	3 (4%)	2 (7%)	19 (6%)
	More than 60 credits	10 (12%)	30 (55%)	6 (11%)	6 (7%)	4 (15%)	56 (19%)

The second set, Data 2, was collected among seven faculties: arts, education, law, medicine, pharmacy, theology and science (Table 2). According to the above-mentioned university statistics, the invitation to participate was sent to approximately 1,920 staff members. A total of 273 teachers filled in the online questionnaire in May/June 2020. Eight of the respondents did not give their consent for their responses to be used in the study, hence the final sample size was 265. The approximate response rate was 14 per cent.

**Table 2:** Data 2: descriptive information on the demographic variables according to the academic field

Data 2		Humanities	Educational Sciences	Law	Medicine	Pharmacy	Science	Theology	Total
		n (%)	n (%)	n (%)	n (%)	n (%)	n (%)	n (%)	n (%)
Gender	Female	57 (65%)	6 (75%)	3 (23%)	30 (59%)	9 (53%)	22 (31%)	10 (59%)	137 (52%)
	Male	27 (31%)	2 (25%)	4 (31%)	18 (35%)	8 (47%)	47 (66%)	7 (41%)	113 (43%)
	Other/missing	4 (5%)	0 (0%)	6 (46%)	3 (6%)	0 (0%)	2 (3%)	0 (0%)	15 (6%)
	Total	88	8	13	51	17	71	17	265
Age	20–30 years	1 (1%)	0 (0%)	1 (8%)	1 (2%)	2 (12%)	4 (6%)	0 (0%)	9 (3%)
	31–40 years	15 (17%)	1 (13%)	2 (15%)	9 (18%)	6 (35%)	30 (42%)	2 (12%)	65 (25%)
	41–50 years	33 (38%)	2 (25%)	2 (15%)	13 (26%)	5 (29%)	20 (28%)	6 (35%)	81 (31%)
	51–60 years	26 (30%)	4 (50%)	5 (39%)	15 (29%)	3 (18%)	11 (16%)	7 (41%)	71 (27%)
	over 61 years	13 (15%)	1 (13%)	1 (8%)	13 (26%)	1 (6%)	5 (7%)	2 (12%)	36 (14%)
Pedagogical training	None	15 (17%)	0 (0%)	6 (46%)	9 (18%)	1 (6%)	23 (32%)	0 (0%)	54 (20%)
	1–10 credits	21 (24%)	0 (0%)	0 (0%)	12 (24%)	7 (41%)	20 (28%)	3 (18%)	63 (24%)
	11–25 credits	23 (26%)	0 (0%)	6 (46%)	20 (39%)	7 (41%)	15 (21%)	3 (18%)	74 (28%)
	26–59 credits	9 (10%)	1 (13%)	0 (0%)	4 (8%)	1 (6%)	8 (11%)	3 (18%)	26 (10%)
	60-credit study module	9 (10%)	0 (0%)	0 (0%)	4 (8%)	0 (0%)	5 (7%)	2 (12%)	20 (8%)
	More than 60 credits	10 (11%)	7 (88%)	0 (0%)	2 (4%)	1 (6%)	0 (0%)	6 (35%)	26 (10%)

The two questions relating to the added value of using digital tools in teaching and learning analysed in this study were: 1) What added value do you think your students derive from your use of digital tools in teaching? 2) What added value do you derive from using digital tools in your teaching? They were part of a more extensive questionnaire that included 10 open questions (Data 1) and six open questions (Data 2).

### Analysis

For the most part, we used the ATLAS.ti Windows (Version 9.1.7.0) in our qualitative analysis of the two data sets because of its versatile coding and analysis tools (ATLAS.ti, 2022). With regard to Data 1, of the 303 teachers, 198 (65%) answered the question about student benefits, and 193 (64%) answered the question about teacher benefits; the respective figures relating to Data 2 were 176 out of 265 (66%) respondents and 148 out of 265 (56%) respondents. Thus, all in all we analysed the responses of 715 teachers. Each respondent mentioned one or several benefits of using digital tools in teaching and learning.

The process followed the general principles of empirically based inductive content analysis (Patton, 2002; Robert, 2016). In other words, the researchers’ reasoning was directly based on the empirical data (Tuomi & Sarajärvi 2009). The analysis, which proceeded in eight phases, started from Data 1: (1) we read several times through the teachers’ answers to the questions “What added value do you think your students derive from your use of digital tools in teaching?” and “What added value do you derive from using digital tools in your teaching?”; (2) we coded different descriptions of added value/benefits from the data; (3) we classified similar types of description of added value/benefits into 20 categories (codes), which (4) we named (20 codes); (5) we reduced the main categories from the original codes (5 main categories). Moving on to Data 2 (6), we coded the data using the original 20 codes from Data 1, adding more when necessary (3 extra codes); (7) an author who had not been involved in the earlier analysis reduced the 23 codes to new main categories (which were almost identical); (8) we discussed the new classification together, resulting in a final output of five main categories and 18 codes, which we describe in the Results section. The respondents’ answers to the two questions were extensively overlapping, hence we decided to classify them using the same emerging categories.

The trustworthiness of the content analysis of the qualitative open-ended data was underpinned by a confirmability criterion (Denzin & Lincoln, 2000; Lincoln & Guba, 1985; Robert, 2016). By confirmability here we mean that the results are based on data rather than on the conceptions of the researchers. To strengthen the confirmability the authors checked and discussed the phases of content analysis several times among themselves. In both rounds, we coded and formed the main categories in separate phases, jointly making comparisons and negotiating outcomes.

Table 3 below gives examples of how we classified some original quotations into the appropriate sub-category (Developing work-related skills), and further into the main category (Enhancing learning).

**Table 3:** Developing work-related skills: classification into the sub-category

Original quotation	Sub-category	Main category
Readiness to face technologies. (38:1)	Developing of work-related skills	Enhancing learning
Courage to use similar and new digital tools in their own teaching. (41:3)		
The digital environment is the current standard while studying and once you enter the workforce. (62:1)		
Similar tools are used in working life in the field in which I teach and different processes are mostly based on their use. (91:1)		
It gives my students the chance to use digital tools themselves as future teachers. (190:1)		
Connection to real life (230:3)		

We used Fisher’s exact test (Field, 2009, 690) in the last phase of the analysis to assess the statistical significance of the differences between academic fields before and during Covid-19. In this phase we used the SPSS procedure.

## Results

### University teachers’ perceptions about the benefits of using digital tools for learning and teaching

According to these university teachers, digital tools offer several benefits for teaching and learning. We reduced these benefits to the following four main categories: 1) practical and administrative benefits, 2) enhancing learning, 3) developing teaching and 4) independence of time and place; we also added one category, namely no benefits (Table 4).

**Table 4:** The benefits of using digital tools in teaching and learning

The main categories and their subcategories	Percentage of responses, before Covid-19	Percentage of responses, during Covid-19
*Practical and administrative benefits* - Sharing and the availability of teaching materials - Instructing, informing and communication - Paperless and other ecological benefits - Efficiency, flexibility and easiness - Submitting and receiving assignments - Course administration and coordination	48%	31%
*Enhancing learning* - Increasing the quality of learning and studying - Activating the student - Making the study process visible - Taking different learners into account - The development of work-related skills - Supporting/utilising collaboration and communality	23%	21%
*Developing teaching* - The development of teacher competence - Improvement in the quality of teaching - Improvement in the quality of teaching materials - Editing and using teaching materials - The meaningfulness of assessment and giving feedback, technical benefits	19%	18%
*Independence of time and place* - Independence of time and place	6%	23%
*No benefits* - Negative/just complicating teaching and learning - No benefits	4%	6%

The first main category, *Practical and administrative benefits* (see Table 4) is the biggest of the four, and it includes all comments related to the effectiveness and easiness of a digital learning environment in terms of reaching students, sharing learning materials and sending and receiving learning assignments. Digital tools are perceived to make course administration easier. This category was stronger before Covid-19 (48% of all open-ended responses) than during the pandemic in spring 2020 (31%).

The second main category is *Enhancing learning*. The teachers felt that digitalisation made it possible to improve the quality of learning and studying, to activate student learning and to make the study process visible. They also felt that using digital tools in teaching and learning made it easier to take different learners into account and thereby to enhance their learning. Using digital tools to support student collaboration was another aspect that was mentioned as being beneficial to learning. Furthermore, some teachers thought that using digital tools in teaching and learning helped their students to acquire work-related skills for the future. The response percentages remained at about the same level during the research period (23% pre-Covid-19 and 21% during the pandemic). The third category, *Developing teaching*, includes comments related to teaching or teaching materials. The teachers felt that digitalisation had developed their teaching, manifested, for example, in higher levels of competence related to using digital tools, and to the quality of their teaching in general. They also found that digital tools motivated them to improve their teaching materials, which in turn enhanced their teaching and helped them to develop as teachers (pre-Covid-19 19%; during the pandemic 18%). The fourth category comprises teachers’ responses related to *Independence of time and place* in teaching and learning. They valued being able to extend teaching and learning processes beyond the formal sessions (pre-Covid-19 6%; during the pandemic 23%). When we compared the teachers' experiences before and during the Covid-19 pandemic we found that the biggest category comprised the practical and administrative benefits in both periods – although to a greater extent pre-Covid-19. Of the other categories, independence of time and place was mentioned more often during Covid-19 (see Table 4).

In the opinion of some teachers, digitalisation either had no benefits or had a negative effect on teaching and learning (pre-Covid-19 4%, during the pandemic 6%).

### Disciplinary differences in teachers’ perceptions of the benefits of using digital tools

**Fig. 3:**
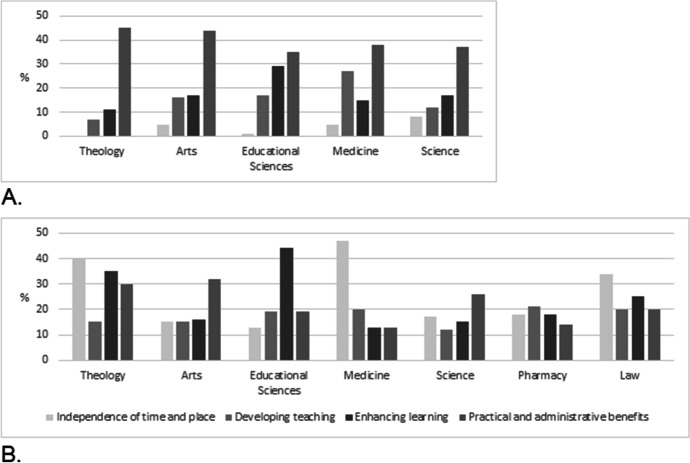
Disciplinary differences in perceptions of online teaching pre-Covid-19 (A.) and during (B.) the pandemic

In line with the second sub-question, we explored the perceptions of teachers in the different academic fields. The findings show that, pre-Covid-19, clearly the most common reason for using digital tools in teaching and learning in all five academic fields related to their practical and administrative benefits (see Fig. 3A). The use of these tools was voluntary at the time, and an additional teaching option, which is evident in the way they tended to serve technological purposes such as sharing teaching materials or course administration. The only exception to this trend was among teachers in the faculty of educational sciences, who referred to enhanced learning as a strong benefit. In addition, teachers in the faculty of medicine valued developing teaching more than teachers in other faculties. The situation changed considerably during the Covid-19 pandemic with the forced digital leap, when disciplinary differences and pedagogical needs were mentioned (Fig. 3B). Two more academic fields were included in this phase, in addition to the earlier five. ‘Independence of time and place’ was more strongly present than earlier in every academic field, and particularly highlighted in two faculties, namely theology and medicine: the teachers experienced this aspect as a major benefit in the sudden and forced pandemic situation. It was also strongly emphasised in the law faculty, which was only included in Data 2. On the other hand, ‘practical and administrative benefits’ weakened compared to the pre-Covid-19 situation, especially in the faculties of arts and medicine. The other clear difference between the pre- and post-Covid-19 situations was in ‘enhancing learning’, which was highlighted in the faculty of theology and stayed strong in educational sciences. In addition to emphasising the possibility to teach and learn despite the pandemic, independently of time and place and by means of digital tools, the teachers prioritised the quality of learning in the difficult situation.

We used Fisher’s Exact Test (FET) as a non-parametric test to assess the statistical significance of the differences between academic fields before and during the Covid-19 pandemic. The benefit variables were classified in two categories: 0 = no benefit; 1 = some benefit. The results reveal significant differences in perceptions of ‘independence of time and place’ described above before and during Covid-19 in the faculties of theology (p < 0.001), arts (p < 0.001), medicine (p < 0.001), educational sciences (p < 0.05) and science (p < 0.05). There was also significant difference in perceptions of ‘enhancing learning’ in the faculty of theology (p < 0.05), as well as in ‘practical and administrative benefits’ in the faculties of arts (p < 0.01) and medicine (p < 0.01) Table 5 below reports the perceived benefits of using digital tools in teaching and learning before Covid-19 and during the pandemic by academic field, as well as the Fisher’s Exact Test scores.

**Table 5:** Benefits of using digital tools by academic field before and during the Covid-19 pandemic

		Before the Covid-19 pandemic	During the Covid-19 pandemic	
		Proportion of codes per academic field (%)	Proportion of codes per academic field (%)	Fisher’s Exact Test (p)
Independence of time and place	Theology	0%	40%	0.001***
	Arts	5%	15%	0.001***
	Educational Sciences	1%	13%	0.039*
	Medicine	5%	47%	0.001***
	Science	8%	17%	0.017*
Developing teaching	Theology	7%	15%	0.415
	Arts	16%	15%	0.931
	Educational Sciences	17%	19%	0.149
	Medicine	27%	20%	0.533
	Science	12%	12%	0.928
Enhancing learning	Theology	11%	35%	0.012*
	Arts	17%	16%	0.787
	Educational Sciences	29%	44%	0.195
	Medicine	15%	13%	0.402
	Science	17%	15%	0.148
Practical and administrative benefits	Theology	45%	30%	0.480
	Arts	44%	32%	0.005**
	Educational Sciences	35%	19%	0.498
	Medicine	38%	13%	0.007**
	Science	37%	26%	0.090

*p < 0.05

**p < 0.01

***p < 0.001

### Teachers’ perceptions of the benefits for students and for teachers

**Fig. 4:**
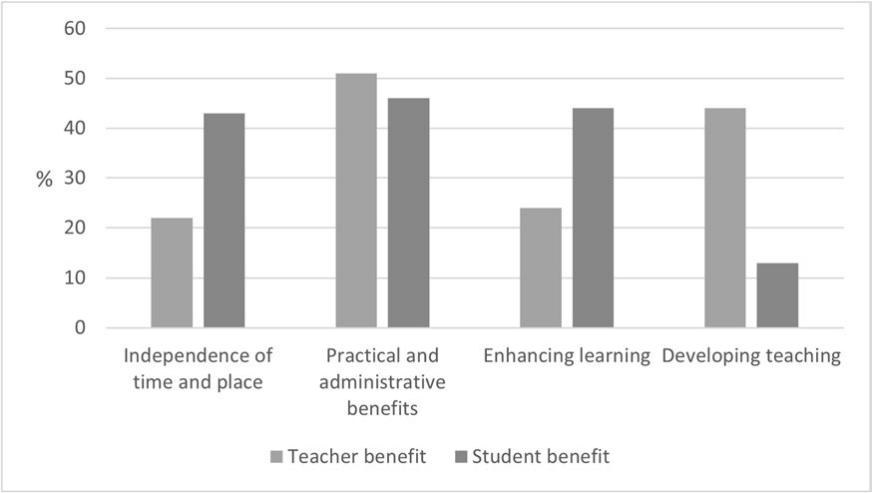
Differences in perceived benefits for teachers and students

In addressing the third sub-question we looked more closely at the intended recipients of any benefits during the period of forced online teaching. In other words, we investigated whether the teachers thought that they were the beneficiaries, or that the use of digital tools was more beneficial to their students. Figure 4 below summarises the findings: the teachers felt that students benefitted more (43%) than teachers (22%) from ‘independence of time and place’ in teaching. For instance, they thought that digitalisation allowed students more flexibility in their studies, and it enabled them to produce teaching materials in advance, such as recordings of lectures, as mentioned in the responses:

*They can see the homework assignments and catch up on the content of the lectures even if they do not attend class. (30:1, Student benefit)*

*If it is not about streaming, you can, for example, make a recording of your lecture when it suits you best, quite a long time in advance. (104:9, Teacher benefit)*

The teachers felt that the ‘practical and administrative benefits’ were almost equal with regard to themselves (51%) and their students (46%). As mentioned below, for example, the accessibility of teaching material benefitted every student, and automatic assessment benefitted the teacher:

*Availability and accessibility, things can be repeated (e.g., grammar videos) (123:1, Student benefit)*

*I can run several extensive MOOC courses in a year when the material is online, and assessment mostly takes place automatically. (12:2, Teacher benefit)*

Teachers emphasised ‘enhancing learning” as one aspect of digitalisation that benefitted students in particular (44%), although it also indirectly benefitted teachers (24%). The respondents described how enriching teaching with different tools enhanced learning, for instance, and how teachers benefitted from digital tools by enhancing communication with students:

*The use of different learning tools gives students more diverse opportunities to familiarise themselves with what needs to be learned, and thus enhances learning. (203:1, Student benefit)*

*I also have more contact with students who otherwise avoid social situations. (21:2, Teacher benefit)*

The teachers also saw ‘developing teaching’ as predominantly a benefit for teachers (44%), but they also recognised that it benefitted students, to some extent (13%). For one teacher the benefit was in enriching future teaching by applying methods that were used in remote teaching. Being forced to teach remotely and to use digital tools widely also inspired them to utilise these tools in contact teaching:

*The opportunities offered by online teaching make me wonder what other forms of teaching I can use besides lecturing. Contact and online teaching could certainly be combined in new ways. (158:7, Teacher benefit)*

In terms of student benefit, for example, the use of digital tools was perceived as a way of helping students to focus on deeper learning such as thinking when they did not have to spend time taking lecture notes:

*Students do not need to take as many notes in lectures as they did before, because the main content is available via PowerPoint in Moodle, which allows them to focus more on thinking and conversation during the lectures. (151:1, Student benefit)*

## Discussion

The focus in this study was on the perceptions of university teachers concerning the use of digital tools in their teaching, in particular on the benefits in terms of teaching and learning before and during the Covid-19 pandemic. According to our findings, teachers perceived four kinds of benefit: (1) practical and administrative and (2) independence of time and place, characterised as practical and technical benefits; (3) enhancing learning and (4) developing teaching, primarily portrayed as pedagogical benefits, related to teaching and learning. Within the TPACK framework, these benefits are positioned at the intersection of pedagogical and technological knowledge, so-called technological pedagogical knowledge (TPK). Upon closer inspection, one could say that practical and technical benefits are positioned closer to technological knowledge, whereas enhanced teaching and learning are located far into the pedagogical side (Fig. 2).

The results are consistent with previous findings implying that digital tools tend to be used for practical purposes (see Table 4) (Amhag et al., 2019; Brady & O’Reilly, 2020; Bond et al., 2018), and in this way reflect more of a teacher-focused approach, as experts have argued (Bond et al., 2018; Marcelo & Yot-Domínguez, 2019). However, the pedagogical benefits we identified were also strong (42% before Covid-19, 39% during the pandemic) which implies the valuing of student-centred learning, as stated in earlier research (Hannafin & Land, 1997; Ottenbreit-Leftwich et al., 2010; Reigeluth, 2014). We also identified a small category in which no benefits of using digital tools in teaching and learning were perceived, or then attitudes were negative (Table 4), which is also reported in Liu et al.’s (2020) research.

Pedagogical benefits referred to performance expectations and social outcome expectations (Bandura, 1986, 1997), indicating a belief that using technology would make someone a more effective teacher, and that technology-aided teaching would result in personal satisfaction. In this sense, our data supported earlier findings reported by Niederhauser and Perkmen (2010) confirming Bandura’s three factors. Interestingly, ‘practical and administrative benefits’ and ‘independence of time and place’ were not clearly related to Bandura’s (1986, 1997) factors, and the social-outcome expectation was not identified in our data. One possible reason for its absence could be that most academic teachers in Finland are quite autonomous in terms of their teaching practices. In addition, there is no practice of compulsory peer observation and feedback among colleagues in Finland, as there is in the UK and Australia (e.g., Wingrove et al., 2018). Hence, the opinions of peers and colleagues may not be as important for them as in Niederhauser and Perkmen’s (2010) study on pre-service teachers. Moreover, there were not many options concerning the use of technology in teaching during the Covid-19 pandemic (when Data 2 was collected) because of the forced digital leap. However, several practical and administrative benefits, such as flexibility and easiness, as well as independence of time and place are in line with the perceived ease of use in Davis’s (1989) acceptance of the information-technology model.

The data sets were collected at two different times: the first before the pandemic when digitalisation was controlled, supported and encouraged but nevertheless voluntary, and the second during it, when teachers were forced to change to online teaching. Interestingly, teachers included in Data 1 referred more often to the practical and administrative benefits of digitalisation, whereas those in Data 2 also emphasised independence of time and place. This may have been because all teaching was being done online due to the pandemic and lockdown (Marinoni et al., 2020), hence the value of teaching and learning was recognised and highlighted regardless of place and time. Digitalisation was encouraged when Data 1 was collected, and training in the use of digital tools was offered to university teachers during the digital leap (Kuuppelomäki et al., 2021). This earlier familiarity with digital tools is also reflected in the teachers’ responses about the use of digital tools before the forced transmission to online teaching (Myyry et al., 2022), which may have affected the extent to which they valued the practical and administrative benefits pre-pandemic. Overall, however, teachers in both time periods recognised the value of digital tools in teaching and learning. One possible explanation for this is that the university offered a lot of support and education with clear institutional guidance during both periods: it has been shown that institutional support and education are vital for teachers when they are transitioning to online teaching and adopting digital tools in their teaching (Naylor & Nyanjom, 2021). Furthermore, there is a connection between the integration of online teaching and both pedagogical and technical support, as well as the institution’s vision of online learning and strong leadership (Rapanta et al., 2020). A shared vision to integrate digital tools into teaching may motivate teachers to change their approach, whereas a lack of commitment to change on the organisational level may inhibit the adoption of digital tools and consequently any change in teaching practices (Tondeur, et al., 2019).

Transmission to forced online teaching during the Covid-19 pandemic highlighted the differences between various academic fields. Although digital tools were previously considered part of a technological continuum of on-site teaching and learning (Baran et al., 2011; Kirkwood & Price, 2014), various forms of pedagogical backing in different academic fields emerged from forced online teaching, based on support and earlier experiences. With regard to the TPACK framework, forced online teaching shifted the focus to the intersection of all three skills, thereby revealing the content-related factors of teaching and learning in higher education and supporting previous findings concerning differences between academic fields (Liu et al., 2020; Marcelo & Yot-Domínguez, 2019). Thus, one could say that, in a way, the change was a blessing in disguise in forcing teachers from different academic fields to develop their teaching in online contexts.

The transfer from on-site to online teaching in the Faculty of Science during the Covid-19 pandemic required the adoption of new technologies in many forms. The blackboard was typically utilised in the on-site teaching of mathematics, physics and chemistry, for instance, and other solutions such as the use of different whiteboards had to be developed for online instruction. Teachers have also been forced to find something to replace typical on-site group instruction sessions and exercises with online real-time events. In pharmacy teaching, for example, versatile activating teaching methods were implemented in mass lectures before the pandemic, and new technological applications were applied to teaching in laboratory work. The transfer to online teaching has required new technical solutions and their implementation to facilitate the teaching. Traditional content-driven lectures given in large lecture halls to the whole-year cohort have been typical in the law faculty, for example. It seems that teachers who focused on the transfer of expert knowledge have sought new technical solutions, thereby embracing independence of time and place in teaching and learning. Studies in the medical faculty, under normal circumstances, follow a strict schedule, for example. They have been traditionally time- and place-dependent, and students’ days on campus have been long. Thus, the opportunity to participate in online distance learning during the pandemic has given students more freedom to organise their own schedules and has shortened their working days. On the other hand, the pandemic affected education in the medical faculty rather differently from other academic fields: lab courses focusing on clinical skills and clinical workplace learning in patient care were continued, but there were smaller groups of students than usual.

Teachers’ pedagogical thinking is reflected not only in the various benefits they see in using digital tools in teaching and learning, but also in how they understand the benefits such tools offer. If the added value of their use is the development of teaching and the benefits that accrue to students, it would seem that the teachers’ pedagogical thinking related to the use of digital tools promotes a student-centred approach to learning. The findings of our study highlight the need for a close dialogue between pedagogical and technological aspects in supporting and educating teachers in higher education to prepare for the digitalisation process (see also Basilotta-Gómez-Pablos et al., 2022; Fernández-Batanero et al., 2020). We therefore offer one response to Jääskelä et al.’s (2017) and Mei et al.’s (2019) calls for wider surveys and a focus on the added value of digital tools in teaching (Jääskelä et al, 2017; Mei et al., 2019). However, there is a need for further research to facilitate deeper-level understanding of the pedagogical thinking among teachers concerning the use of digital tools (Kim et al., 2013).

## Strengths and limitations

This study has methodological strengths and limitations that should be considered in the drawing of any conclusions. A major strength concerns the use of two individual datasets to explore the perceived benefits of digital tools for teaching and learning among university teachers. The response rates for both data sets were somewhat low (Data 1: 18%; Data 2: 14%), thereby raising concerns about the generalisability of the results. However, the sample represented teachers at the target university sufficiently in terms of gender. In any case, sample representativeness is considered more appropriate than response rate as a criterion for evaluating the validity of a study (Cook et al., 2000), and a low response rate is a common phenomenon in e-mail surveys (Shih & Fan, 2009). Data 1 included teachers from five faculties, and Data 2 expanded the sample to include teachers from seven faculties in a large multifield research-intensive university. Thus, we were able to gather rich data from different academic fields and thereby build a more comprehensive understanding of the phenomena. However, this was not a longitudinal study and we do not know to what extent the respondents in the two data sets are the same. Thus, causal conclusions and conclusions about the development of perceptions can only be made on the more general institutional level. We adopted an inductive data-analysis strategy (Patton, 2002), the trustworthiness of the analysis being underpinned by a confirmability criterion (Denzin & Lincoln, 2000; Lincoln & Guba, 1985). The discussions and negotiations between the researchers during the analysis phases increased the reliability of the study. Nevertheless, caution must be exercised in generalising the results to other contexts and settings. More studies are needed to validate the findings and the instrument further. Despite these limitations, however, the study offers significant insights into the perceptions of university teachers about using digital tools and about the added value they bring to their teaching and to student learning.

## Conclusions and practical implications

In sum, our research indicates that teachers perceive both practical and technical as well as pedagogical benefits in using digital tools. Within the technological pedagogical content knowledge (TPACK) framework these benefits can be positioned in opposite edges of the section of technological pedagogical knowledge (TPK). Hence, the results reveal how both teacher-focused and student-centred approaches guide teachers’ choices. In addition, the identified pedagogical benefits fit with performance expectations of the social cognitive theory.

Voluntary and forced digital leaps provided different kinds of consequences in teaching and learning. The forced transmission to the online teaching environment showed the difference between “online teaching” and “emergency remote teaching”: during the controlled digital leap the digital tools generally provided more practical and technological benefits, but they also had positive pedagogical effects. The forced digital leap, in turn, emphasised the value of independence of time and place in teaching and learning during the Covid-19 pandemic on the one hand, and on the other hand, it revealed differences among the academic fields in how teachers experienced the benefits of digital tools. Teachers’ perceptions of the benefits of using digital tools for students and for themselves varied to some degree. On one hand, teachers felt that students benefitted more than teachers from independence of time and place in teaching and enhanced learning that the use of digital tools afforded. On the other hand, teachers felt that they benefitted more than students from developing teaching that the use of digital tools enabled.

Furthermore, as practical implications of this research it can be stated that there is a need for further dialogue between the pedagogical and technological aspects of supporting and educating teachers in higher education for digitalisation. For example, training modules could be developed that combine knowledge of pedagogically well-argued choices in course planning, knowledge of the affordance of various digital tools for teaching and learning situations, and the skills required to use digital tools in a meaningful way: this could help teachers to plan and implement teaching incorporating the use of digital tools in a way that is pedagogically valid. The results indicate that from teachers’ perspective, educational technology should lighten the workload of teachers and be easy to use, but also it should be evident how using digital tools in teaching enhance learning. Finally, there is a need to consider the degree to which the support and training the university offers its teachers should be modified based on the respective academic fields. In conclusion, our research sheds light on the added value of using digital tools in higher education, but there still is a need for more detailed studies on the teachers’ pedagogical thinking behind their use in teaching and learning.

## Data Availability

The datasets generated during and/or analysed during the current study are available from the corresponding author on reasonable request.

## Declarations

### Confirmation of not publishing or submitting elsewhere

The content of the manuscript has not been published or submitted for publication elsewhere.
